# Using publicly available UK datasets to identify recruitment sites to maximise inclusion of under-served groups: three case studies

**DOI:** 10.3310/nihropenres.13551.2

**Published:** 2024-09-13

**Authors:** Alison Booth, Catriona McDaid, Ashley Scrimshire, Harvinder pal Singh, Arabella Scantlebury, Catherine Hewitt

**Affiliations:** 1 Health Sciences, University of York, York, England, YO10 5DD, UK; 2 Academic Centre for Surgery, South Tees Hospitals NHS Foundation Trust, Middlesbrough, England, TS4 3BW, UK

**Keywords:** EDI, inclusivity, datasets, methods, trial, recruitment sites

## Abstract

**Background:**

There is strong evidence that those recruited into studies are not always representative of the population for whom the research is most relevant. Development of the study design and funding decisions are points in the research process where considerations about inclusion of under-served populations may usefully be made. Current practical guidance focuses on designing and modifying participant recruitment and retention approaches but an area that has not been addressed is recruitment site selection.

**Methods:**

We present case studies of three NIHR funded trials to demonstrate how publicly available UK population datasets can be used to facilitate the identification of under-served communities for inclusion in trials. The trials have different designs, address different needs and demonstrate recruitment planning across Trauma centres, NHS Trusts and special educational settings.

We describe our use of national freely available datasets, such as those provided by NHS Digital and the Office for National Statistics, to identify potential recruitment sites with consideration of health status, socio-economic status and ethnicity as well as clinical and risk factors to support inclusivity.

For all three studies, we produced lists of potential recruitment sites in excess of the number anticipated as necessary to meet the recruitment targets.

**Discussion:**

We reflect on the challenges to our approach and some potential future developments. The datasets used are all free to use but each has their limitations. Agreeing search parameters, acceptable proxies and identifying the appropriate datasets, then cross referencing between datasets takes considerable time and particular expertise. The case studies are trials, but the methods are generalisable for various other study types.

**Conclusion:**

Through these exemplars, we aim to build on the NIHR INCLUDE project, by providing trialists with a much needed practical approach to embedding EDI into trial design at the grant application stage.

## Introduction

### Background

In response to the substantial body of evidence that those recruited into research are not always representative of the population for whom the research is most relevant ^1– 4^, there is increasing recognition of the imperative to include under-served communities. In this context under-served communities have been described as groups that are ‘less well represented in research than would be desirable from population prevalence and healthcare burden’ ( https://www.nihr.ac.uk/about-us/our-key-priorities/under-served-communities.htm). More inclusive research, addressing ethnicity, diversity and inclusivity (EDI), supports generalisability of results, and adoption into practice ^5^.

Whether a group is under-served is context and study specific. Groupings for consideration include by: demographic factors (e.g. age, sex, ethnicity, education); social and economic factors (e.g. employment, socio-economic status, geographic location, language, digitally excluded); health status (e.g. mental health condition, cognitive impairment, physical disabilities); and disease specific factors (e.g. rare diseases) ^5^.

The UK National Institute for Health and Care Research (NIHR) and other health and social care research funders want to ensure that the research they fund meets the needs of the whole population for which it is relevant ^6^. The NIHR-INCLUDE project roadmap shows potential points in a trial’s lifecycle where considerations and decisions about inclusion of under-served populations may most usefully be made ^5^. These include development of the study design by researchers through to research funding decisions by panels, all being made with consideration to ensuring inclusion of a study population that reflects the target population. Details of how under-served groups have been identified and showing how trial recruitment will target where the relevant groups are situated, needs to be reported in funding applications if funding panels are to make decisions about whether inclusivity has been appropriately considered.

There are studies that have identified context and condition specific barriers to inclusion of under-served groups in trials and proposed solutions ^7– 9^. Current practical guidance to address these barriers focuses on designing and modifying participant recruitment and retention approaches once a trial is up and running. Available guidance includes consideration of eligibility criteria and the recruitment pathway, participant facing materials, cultural competency and building community partnerships ^10^. An area that has not been addressed is recruitment site selection. Identifying sites that include the relevant under-served population should enhance the chances of their inclusion in a study.

In this paper, we use three NIHR funded trials as case studies for demonstrating how publicly available UK datasets can be used to facilitate the identification of under-served communities for inclusion in trials. Two of the three grant applications were commended by the funding panels for their approach to inclusivity.

### Overview of case studies

The case studies we describe here are of three randomised controlled trials (RCTs), where we piloted use of UK national, freely available datasets to identify recruitment sites with consideration of health status, socio-economic and ethnicity characteristics to support inclusivity of under-served populations. In planning a trial we consider equality, diversity and inclusion across several aspects of the trial design and delivery including the eligibility criteria, the primary outcome measure, public involvement plans, site and participant recruitment strategy. Our focus here is site selection.

The example trials have different methodological approaches, address different needs and demonstrate recruitment planning across both health and social care settings. An overview of the key aspects of the studies is presented in Table 1. All three trials have internal pilots and an economic evaluation; TIDE ( https://www.york.ac.uk/healthsciences/research/trials/ytutrialsandstudies/trials/tide/) and INTERACT ( https://www.york.ac.uk/healthsciences/research/trials/ytutrialsandstudies/trials/interact/) also include qualitative process evaluations. The trials were all funded by the NIHR Health Technology Assessment (HTA) programme. For each of the trials we used the site selection approach with a different level of intensity: i) as the main method of selecting the recruitment sites for the trial (INTERACT), ii) to shortlist/prioritise sites from an existing group of sites we had previously worked with on a related study (TIDE) and iii) to check the suitability of a pre-identified group of recruitment sites (DIDACT) ( https://www.york.ac.uk/healthsciences/research/trials/ytutrialsandstudies/trials/didact/).

**Table 1.** Key aspects of case study trials.

	DIDACT	TIDE	INTERACT
**Full study title**	Surgery compared with sling immobilisation in the management of adults with a displaced fracture of the distal clavicle: a multi-centre, pragmatic, parallel-group, non-inferiority, randomised controlled trial	A multi-centre randomised controlled non-inferiority and cost effectiveness trial comparing Polyhexanide and Chlorhexidine with Neomycin to Mupirocin for nasal meticillin-resistant Staphylococcus aureus (MRSA) decolonisation amongst adult hospital in-patients	Intensive Interaction for children and young people with profound and multiple learning disabilities: INTERACT cluster randomised controlled trial
**Population**	Patients with a radiological diagnosis of a displaced fracture of the distal clavicle	Hospital in-patients identified as being colonised with MRSA	Children and young people with profound and multiple learning disabilities (PMLD).
**Age**	Adults (16 years and over)	Adult (16 years and over)	Children/young people (between 3 and 25 years)
**Intervention**	Sling immobilisation	Nasal polyhexanide gel plus chlorhexidine body wash or nasal chlorhexidine with neomycin cream plus chlorhexidine body wash	Intensive interaction for 18 weeks
**Control**	Surgical fixation	Nasal mupirocin ointment plus chlorhexidine body wash	Care as usual
**Outcome**	Upper extremity disability and symptoms at 12 months (Disability of Arm, Shoulder and Hand (DASH))	Successful early nasal decolonisation (a negative nasal MRSA swab 48 hours after treatment completion)	Communication skills at 32 weeks (Communication Complexity Scale)
**Setting**	Major trauma centres and trauma units	Secondary care hospital sites	Special educational settings and mainstream educational settings with specialist facilities

## Methods used to identify and prioritise study sites

### Pre-identified potential recruitment sites

Standard, pragmatically focussed approaches to identifying sites include using the networks of clinical leaders in their field (as with all three of our case studies), an existing relationship with specific NHS Trusts through a previous study, as we utilised with TIDE ^11^, or a survey of potential Principal Investigators to gauge level of interest. The latter approach identified a high level of interest from educational settings and speech and language therapists in the INTERACT trial. Likewise, a national survey of British Elbow and Shoulder Society (BESS) surgeons, secured expressions of interest from 23 sites to be involved in the DIDACT trial. However, we were aware that we had identified the sites based on level of interest from the clinical teams and before prioritising these sites for recruitment we needed to have a fuller understanding of whether recruiting from these sites would facilitate recruiting participants reflecting the range of people who have a distal clavicle fracture. The same principle was applied to the pre-existing list of NHS Trusts for TIDE, and the interest from educational settings for INTERACT.

### Identifying risk factors

The next step therefore was to collate information on the epidemiology of the condition to fully understand the target population. For DIDACT, the information available was very sparse. Clinical experience is that this fracture can be experienced by anyone but it is a more common injury amongst younger physically active people, particularly men of working age ^12^. For TIDE, our literature searches found a systematic review which identified risk factors associated with increased risk of the clinical problem being addressed by the trial – specifically risk factors of MRSA colonisation ^13^. Of particular interest to our study were antibiotic use, previous MRSA colonisation, and care and nursing home residents. For INTERACT we found that over 10,000 children and young people in England have PMLD. All have multiple disabilities, the most significant being profound intellectual disability and great difficulty communicating. Higher rates of PMLD have been found among Traveller children of Irish heritage, Pakistani, Bangladeshi, ‘other’ Asian and ‘other’ black heritage and ‘any other’ ethnic group ^14^ ( https://www.gov.uk/government/publications/people-with-learning-disabilities-in-england/chapter-1-education-and-childrens-social-care-updates).

### Selecting datasets and cross referencing

Having established key identifying features for potential sites and participants, we then applied these criteria to relevant accessible datasets. In Table 2 we set out a simple overview of the drivers for site selection identified in the epidemiological preparatory work, and the sources subsequently used to find potential study sites to support inclusivity. Box 1 contains details of the sources used. Depending on the identified trial specific features and the EDI factors to account for, where necessary an order of priority for searching and cross referencing was used. In addition, pragmatic accommodations to facilitate cross referencing between datasets was necessary. For example, the names of NHS Trusts had to be linked to their Clinical Commissioning Group (CCG) before the lists could be applied to the index of multiple deprivation dataset. All the searches used the most up to date information available in each database and for each field.

**Table 2.** Drivers for recruitment site selection and sources used for identifying potential sites.

	DIDACT	TIDE	INTERACT
**Drivers for ** **study site ** **selection**	**Clinical:** distal clavicle fracture **Risk factors**: young males **EDI:** Aim to identify ethnically diverse and socially deprived recruitment sites	**Clinical:** NHS Trusts with high rates of MRSA **Risk factors**: antibiotic use; previous MRSA colonisation; care/ nursing home residents **EDI:** Trusts with most deprived areas, and highest proportion of ethnic minorities areas	**Clinical**: Profound and Multiple Learning Difficulty (PMLD) **Risk factors:** Traveller children of Irish heritage, Pakistani, Bangladeshi, ‘other’ Asian and ‘other’ black heritage and ‘any other’ ethnic group **EDI:** Prioritise inclusion of schools in areas of deprivation and ethnic minorities with students between age 3 to 25
**Data sources ** **used**	Office for National Statistics. Clinical Commissioning Group population estimates mid-2019 NHS Digital. Ethnicity categories using General Data Protection Regulation (GDPR) and Health Episode Statistics (HES) datasets by Clinical Commissioning Group 2020 NHS Digital. Clinical Commissioning Group Outcome Indicator Sets (CCG OIS) Summaries Ministry of Housing Communities & Local Government. English indices of deprivation 2019.	Public Health England. Fingertips public health data NHS. Find your local Clinical Commissioning Group. NHS Digital. Geographic search NHS Digital. Clinical Commissioning Group Outcome Indicator Sets (CCG OIS) Summaries Ministry of Housing Communities & Local Government. English indices of deprivation 2019.	Education Statistics. Special educational needs in England (2021/22) Ministry of Housing Communities & Local Government. English indices of deprivation 2019.

For this step, the process used for each of our case studies is described below. See Box 1 for the sources used in each case.

Box 1. **Sources used**

Details and links to publicly available datasets used in our three case studies.

**Table:** 

**Public Health England. Fingertips public health data** Fingertips is a large public health data collection complied by the Office for Health Improvement and Disparities. Data are organised into themed profiles for example – Antimicrobial resistance local indicators. https://fingertips.phe.org.uk
**NHS. Find your local Clinical Commissioning Group (CCG)** Formerly provided a list of CCGs, now listing Integrated Care Boards. ( https://www.nhs.uk/service-search/other-services/Clinical%20Commissioning%20Group/LocationSearch/1)
**NHS Digital. Geographic search** Search for geographically responsible Local Health Authorities, Higher Health Authorities and Former CCG / Sub Integrated Care Board (ICB) Locations within England or Wales using a full or partial postcode. ( https://odsportal.digital.nhs.uk/Geographic/Search)
**NHS Digital. Clinical Commissioning Group Outcome Indicator Set (CCG OIS) Summaries** Index of multiple deprivation rankings and deciles are produced by CCGs. CCGs are NHS organisations set up by the Health and Social Care Act 2012 to organise the delivery of NHS services in England. CCGs have boundaries that are coterminous with those of Lower-Layer Super Output Areas (LSOAs). https://digital.nhs.uk/data-and-information/publications/statistical/ccg-outcomes-indicator-set
**Ministry of Housing Communities & Local Government. English indices of deprivation 2019.** The Index of Multiple Deprivation 2019 is a measure of multiple deprivation at the small area level and a key dataset in all our searches. It includes income deprivation, employment deprivation, education, skills and training deprivation, health deprivation and disability, crime, barriers to housing and services and living environment deprivation. https://www.gov.uk/government/statistics/english-indices-of-deprivation-2019
**Education Statistics. Special educational needs in England (2021/22)** This publication combines information from the school census (state-funded schools), school level annual school census (independent schools) and general hospital school census on pupils with special educational needs (SEN). The publication includes breakdowns by type of SEN provision, type of need, age, national curriculum year group, gender, ethnicity, English as a first language and free school meal eligibility. https://explore-education-statistics.service.gov.uk/find-statistics/special-educational-needs-in-england
**Office for National Statistics. Clinical commissioning group population estimates mid-2019** Size, age, sex, and geographic distribution of the UK population, including data on international migration, migration within the UK, changes in the population and the factors driving these changes. Datasets produced by ONS from census data. Annual population estimates. Figures are available for various administrative and electoral geographies and for different population sub-groups, for example, estimates of the very old and estimates by marital status. Figures are produced by CCGs. https://www.ons.gov.uk/peoplepopulationandcommunity/populationandmigration/populationestimates/adhocs/12429clinicalcommissioninggroupsccgspopulationestimatesbysingleyearofageandsexenglandmid2001tomid2019
**NHS Digital. Ethnicity categories using General Data Protection Regulation (GDPR) and Hospital Episode Statistics (HES) datasets by CCG 2020** NHS Digital collects information on ethnicity in areas covering births, general practice, and secondary uses. It maintains an ethnic category information asset that combines data from general practice and hospital episode statistics (HES) with coverage of 88%. https://digital.nhs.uk/data-and-information/publications/statistical/mi-ethnic-category-coverage/current


*N.B. Some of the links include access to the latest and previous releases and some also now reflect the change from CCGs to ICBs.*

### TIDE

For TIDE, the search started in Fingertips public health data for any relevant or related data fields as identified in the systematic review, also for additional recognised markers of MRSA colonisation used by this national dataset. For some items, proxies or surrogate markers had to be used due to the limitations of publicly available national datasets. For example, we searched on bacteraemia rates as a proxy for previous MRSA colonisation. This data source gave a value for each of four clinical indicators, for example 147.3 prescribed antibiotics per 1000 registered patients per quarter by CCG. See Table 3 for all markers used.

**Table 3.** Surrogate/proxy markers used for variables in TIDE.

Variable	Surrogate/proxy used
MRSA colonisation	Bacteraemia rates
Area of deprivation	Postcode
Antibiotic use	Total number of prescribed antibiotic items per 1000 registered patients by quarter
Patients living in a long-term care facility	Care home beds per 100 people Nursing home beds per 100 people

Each NHS Trust was matched to their corresponding CCG and the value for each indicator mapped to each NHS Trust. For Trusts serving multiple CCGs the mean value for each indicator was calculated. For example, if Trust A is covered by CCGs B and C which had antibiotic prescribing rates of 80 and 100 per 1000 patients, then a rate of 90 was used as the antibiotic prescribing rate for Trust A. It was assumed each CCG carried an equal weight within each Trust. NHS Trusts were then ranked from highest to lowest for each clinical indicator i.e. most antibiotics prescribed, to least. The ranking of the four clinical indicators within each Trust were then combined to give a combined risk of the clinical problem (i.e. combined MRSA colonisation risk) for each NHS Trust.

In a similar manner to the clinical indicators, indicators for underserved populations were searched for in publicly available national datasets. In this instance three indices of deprivation from the ONS and one measure of ethnicity from census data were identified and used. For the indices of deprivation used, each CCG was ranked nationally, most deprived to least. For ethnicity a combined non-white proportion was calculated for each CCG. CCGs were then ranked from highest proportion of non-white population (most diverse) to lowest. The non-white groupings were, Mixed/multiple ethnic, Asian/Asian British, Black/African/Caribbean/Black British and Other ethnicity groupings. Ranking of CCGs by each of the different categories would have been possible had we been seeking to include a specific ethnic group.

These data were not available by NHS Trust but instead by CCG, so the Trust mapping to CCGs was used again. The rankings for each of the four indicators for underserved populations were then merged to give a combined ranking for underserved populations for each Trust. In addition, clinical and underserved population rankings were also combined to give a score for clinical risk and degree of underserved population for each NHS Trust.

### INTERACT

With INTERACT, we found routinely available national data for pupils with Special Educational Needs in England, which includes breakdowns by type of SEN provision, and then we identified those schools including children/young people with PMLD. This school level data was combined with other national datasets that included data on ethnicity, index of multiple deprivation, age and gender.

Datasets were combined using the local authority code as that identifier was common across all datasets. Most datasets were from 2021/22 but the index of multiple deprivation dataset was only available from an earlier time point (2019), and the data could only be linked at the local authority level. Schools were ranked based on the combination of high PMLD numbers, low deprivation, and high percentage of ethnic minority groups.

After ranking the schools, we explored the age spread by school. Only a small percentage of state special educational settings include people over 19 and it was felt important to include the full range of the school population where it existed. Hence, we checked whether the schools we ranked to target for recruitment included over 19-year-olds.

### DIDACT

For DIDACT we collated the most up to date regional level data from multiple national sources to identify areas with high proportions of young males, alongside the index of multiple deprivation, index of health deprivation and ethnicity. Although there was no specific epidemiological data of the injury being more prevalent in particular socio-economic or ethnic groups, we linked and combined the data on proportion of males with data on ethnicity and deprivation to address the broader concern of ensuring the trial recruited from geographic populations with high disease burden which have been historically underserved by research activity.

Initially we matched each site to CCGs that provided us with an identifier to merge with the health deprivation, age, gender and ethnicity data. We calculated the percentage of the population that were male in the 16–25 age group and combined non-white backgrounds and for each we ranked them across all CCGs. The percentage of interested sites that fell into the top 5%, 10% and 20% nationally for clavicle fractures (young males 16–25) and collectively for the most vulnerable and underserved populations (ethnicity; health deprivation) were calculated. For the combined variables we assumed that variables were equally weighted.

Datasets were combined from different time periods and not all CCGs were available for all variables of interest. These were matched as closely as possible and changes in CCGs were matched as closely as possible using the linked variables within the datasets where available.

### Search results and pre-identified potential sites

For DIDACT, examination of the data showed that the 23 sites already identified through a survey of surgeons included one in three of the top 5% of regions with the most vulnerable and underserved populations, and just under half of the top 10% of regions at the highest risk from a clavicle fracture. We thought that this was a reasonable selection of sites, also taking into consideration enthusiasm from the clinical teams.

With TIDE the scores were mapped against a list of NHS Trusts who we had successfully worked with previously to identify the 10 Trusts we planned to target first for recruitment in the pilot phase.

For INTERACT, given this is an under-researched area with no established networks of schools taking part in research, we did not have access to high recruiting sites and thus we used this list of schools as our sampling frame of schools to contact for participation. We created a list of approximately 400 schools serving the target population.

For all three studies, we produced lists of potential recruitment sites in excess of the number anticipated as necessary to meet the recruitment targets.

## Discussion

To counter the evidence showing that research often excludes groups for whom it is relevant, we explored ways to identify study recruitment sites to improve inclusivity for our trials. The three case studies we present investigate different interventions, in different settings, covering both health and social care. We reflect here on the challenges to our approach and some potential future developments.

When identifying potential study sites, preparatory work to identify the key search parameters is essential to aid efficient and accurate identification of data sources and understanding the data they contain. For TIDE we had quite rich epidemiological information to inform which populations we needed to target and hence which datasets to access. Less so for the DIDACT study, which evaluated treatment for a fracture for which there is limited epidemiological data but, broadly speaking, could happen to anyone. Here we took the broader approach of identifying sites in areas with more ethnically diverse populations and deprivation on the assumption that this would situate the study among diverse communities. We found that the study setting affected complexity. The task was more straightforward for DIDACT, where participants could only be identified in NHS major trauma centres and trauma units, and TIDE where potential participants had to be NHS hospital in-patients. For INTERACT, considerations were more complex as children and young people with special educational needs attend different types of settings based within local authorities. In addition, not all educational settings serve children and young people with PMLD and those that do vary in the age range catered for.

We recognise that the use of the national datasets as in our case studies is not perfect. We had to use proxies such as, hospital rates of bacteraemia instead of previous MRSA colonisation of an individual and the use of postcode is a crude measure of area of deprivation. Data from the sources were not always current and this was compounded by the data not yet reflecting the organisational changes in the NHS from CCGs to ICBs. This created a challenge for cross matching, as seen where NHS Trusts had to be matched to CCGs to identify some of the necessary data. However, the approach enabled us to move beyond an even blunter approach of thinking about site location in broad regional terms and we were able to demonstrate to the funders that we had considered and made efforts to address the question of ethnicity, diversity and inclusivity. We would have liked to use person-level data such as HES data rather than the population level data available in publicly available datasets. However, currently the complexities, time and cost of accessing person-level datasets prohibit use at the grant application stage. Hopefully more accessible and sophisticated approaches will become possible over time. We await the outcome of the Sudlow review, ‘Unifying Health Data in the UK’ ( https://www.hdruk.ac.uk/helping-with-health-data/the-sudlow-review/), expected Spring 2024, with interest. The review is looking at health related data across the UK, with a view to seeing how data can be better managed and to identify barriers to the safe and secure linkage and analysis of data from different sources for public benefit.

Two of our funding applications (TIDE and INTERACT) were for commissioned funding calls, with a relatively limited period to develop the research plan and application. An advantage of the approach was that it was feasible within the time-frame available: the data utilised were readily available, with no fee to access, and did not require any access permissions. Although the method was feasible to use, it had an opportunity cost as it required researcher time that could have been utilised differently. The activity related to understanding the target population for each study, including meetings with health and care professionals, patient and public involvement groups and searching the literature, would have happened anyway as that informed other strategies related to inclusive study design. The main additional time related to identification of the appropriate data sources, collating and merging, then checking. This ranged from two to five days related to experience with handling data and the software used.

There are other important considerations for selecting recruitment sites for a trial, in particular expected recruitment success at a site i.e. recruiting to target and inclusion of sites based on their expected recruitment performance. Although prediction of sites that will successfully recruit to a trial is not an exact science ^15^, we implicitly took this into consideration by starting with a group of sites we had previously worked with (TIDE) or based on surgeon interest (DIDACT). This approach may have created some disadvantage to sites currently underserved by research. For those sites we included, in theory less experienced sites and sites with less infrastructure may be slower to open to recruitment and therefore recruit for less time during the study offering less opportunity for underserved communities to take part in the research.

There is evidence that in large multi-centre trials most of the participants come from a small number of high recruiting sites ^16, 17^. In addition, recruiting at a site serving a diverse population does not necessarily mean that a diverse population will be recruited to the study. Therefore, we plan to review the success of our site selection strategy at the end of the trials by evaluating the demographic profile of recruiting sites, time open to recruitment, number recruited and the characteristics of the study population.

The examples we provide were for RCTs, but the methods are we believe generalisable for various other study types undertaken within the UK. Hurtado-Chong *et al.* offer an alternative approach which they piloted for an international cohort study ^18^. Their ‘standardised, objective multistep method’ involved inviting expressions of interest from an international network of clinicians. While they found their approach encouraging, it is hard to see how under-served populations were accounted for. In an observational study to see if health research in England between 2013 and 2018 was undertaken where the burden of disease was the greatest, Bower et al found that geographical variations in recruitment did not reflect the suitability of the population for research ^19^. They called for the development of indicators ‘to assess the fit between research and need, and to allow assessment of interventions among funders, researchers and patients to encourage closer alignment between research activity and burden.’ Likewise, one of the aims of the INCLUDE project was to ensure that representation of under-served groups is a consideration in funding decisions, regulatory approvals and policies ^5^. The need to make sufficient resources available was also recognised. This work is now being taken forward as part of the NIHR Under Served Communities programme ( https://www.nihr.ac.uk/about-us/our-key-priorities/under-served-communities.htm)

The potential for synergies between electronic health records and data of value in the planning, conduct and measuring impact of clinical trials prompted NIHR, Health Data Research UK (HDR UK) and the Clinical Practice Research Datalink (CPRD) to discuss ways to accelerate the agenda for ‘data-enabled clinical trials’ ^20^. They highlight the value that electronic health records could have for assessment of feasibility, particularly where complex pathways are involved. The use of electronic health records, where available, could give a more realistic estimate of actual numbers of potentially eligible participants. For primary care datasets this could almost be in ‘real’ time; the current lag in availability of secondary care data is likely to remain for now. However, at present accessing such data is costly and time-consuming.

How our method works in practice in terms of identified sites agreeing to take part and then whether the participants recruited fulfil our aspirations for true inclusivity remain to be seen. Also, it should not be utilised in isolation from other methods and approaches to how research is designed to promote inclusivity. Thought still needs to be given to issues such as avoiding unjustified exclusion criteria and taking inclusive approaches to recruitment and retention ^9, 10^.

The datasets we accessed are all free to use but each has their limitations. Agreeing search parameters, acceptable proxies and identifying the appropriate datasets, then cross referencing between datasets takes considerable time and particular expertise. Through these exemplars, we aim to build on the NIHR INCLUDE project, by providing trialists with a much needed practical approach to embedding EDI into trial design from the grant application stage.

## Data Availability

Please see Box 1 for the sources used in each case.
