# Supplementary material for: Using publicly available UK datasets to identify recruitment sites to maximise inclusion of under-served groups: three case studies
Source: NIHR Open Res. 2024 Sep 13;4:18. Originally published 2024 Apr 12. [Version 2] doi: 10.3310/nihropenres.13551.2 (PMC11320047; doi:10.3310/nihropenres.13551.2)
Supplement: Peer review history

# Reviewer report: Reviewer response for version 2

**DOI:** 10.3310/nihropenres.14928.r32103

**Published:** 2024-10-14

**Authors:** Maria Florez

**Affiliations:** 1 Tufts University, Medford, Massachusetts, USA

Thank you for the opportunity to review this article.

 The article is nicely written. It presents results from a study aiming to build on NIHR INCLUDE by offering a new approach for embedding equity, diversity, and inclusion principles into clinical study design beginning at the grant application stage and using publicly available data to inform site selection. The authors include three RCTs as examples on how to achieve this.

 The article explains the rationale for developing a new method to address representation in clinical trial participation by improving site selection. The authors explain that study funding, design and planning are crucial points at which decisions that affect inclusion of underserved populations are made. The authors may consider strengthening the argument about the importance of site selection for diversity and inclusion in clinical research by citing additional literature on the relationship.

 The authors opt for a case study methodology. They present three unique cases of NIHR-funded trials in the UK that use public data sources to inform site selection. The authors are commended for including cases that represent a wide array of trial designs, conditions, and site settings. The authors outline a process that includes pre-identifying potential recruitment sites, identifying risk factors, selecting datasets, and cross-referencing. The authors adequately define the races/ethnicities included in the “non-white” category where applicable and provide sufficient details to allow for replication by clearly stating the location of source datasets and the steps taken in each example.

 The authors offer a practical approach for embedding equity and inclusion into clinical trial design, while noting its limitations.

Is the rationale for developing the new method (or application) clearly explained?

Yes

Is the description of the method technically sound?

Yes

Are the conclusions about the method and its performance adequately supported by the findings presented in the article?

Yes

If any results are presented, are all the source data underlying the results available to ensure full reproducibility?

Yes

Are sufficient details provided to allow replication of the method development and its use by others?

Yes

Reviewer Expertise:

Drug development (including clinical research optimization;  site strategies;  inclusivity in clinical trials; and digital transformation in drug development).

I confirm that I have read this submission and believe that I have an appropriate level of expertise to confirm that it is of an acceptable scientific standard.

# Reviewer report: Reviewer response for version 1

**DOI:** 10.3310/nihropenres.14710.r32016

**Published:** 2024-08-01

**Authors:** Sarah Kobayashi

**Affiliations:** 1 University of Sydney, Sydney, Australia

Thank you for allowing me to review this manuscript. It is a well-written manuscript, addressing a real-world issue that we have in clinical research. It is refreshing to see this kind of work being done and is much needed, particularly as RCTs are becoming larger and results are being generalised at a population level.

 The authors highlighted three different clinical RCT case studies where they leveraged from publicly available datasets to help identify communities to target for more inclusive trials. The mapping approach in Table 2 of identifying which publicly available dataset to use for which study can be generalised to researchers in other countries but may be limited by the availability and accessibility of datasets outside of the UK. Regardless, the methodology is sound and relatively replicable.

 In the discussion, the authors described challenges, strengths and limitations - all of which are helpful for readers to understand how they can replicate these approaches in their own research setting. 

 Overall, it was a pleasure to read this manuscript!

Is the rationale for developing the new method (or application) clearly explained?

Yes

Is the description of the method technically sound?

Yes

Are the conclusions about the method and its performance adequately supported by the findings presented in the article?

Yes

If any results are presented, are all the source data underlying the results available to ensure full reproducibility?

No source data required

Are sufficient details provided to allow replication of the method development and its use by others?

Yes

Reviewer Expertise:

My area of research is in clinical trials in osteoarthritis management programs. I'm starting a program of research in under-represented communities in my area, so this is very interesting and relevant!

I confirm that I have read this submission and believe that I have an appropriate level of expertise to confirm that it is of an acceptable scientific standard.

# Response

**Published:** 2024-09-07

**Authors:** Alison Booth

**Affiliations:** Health Sciences, University of York, York, England, UK

Thank you for your supportive review, we are particularly pleased that you feel our approach could be used in non-UK settings.

# Reviewer report: Reviewer response for version 1

**DOI:** 10.3310/nihropenres.14710.r32022

**Published:** 2024-06-20

**Authors:** Megan Wood

**Affiliations:** 1 University of Leeds, Leeds, England, UK

The manuscript describes a study which aims to show how publicly-available datasets can be used to improve inclusivity and diversity in clinical trials, with a focus on the recruitment site. The authors use three RCTs as exemplars for how this can be done.

 The authors should be commended for a very thorough and well-written article which provides clear examples on how to improve diversity in clinical trials across health and social care. 

 The authors provide a well-justified rationale for why it is necessary to consider EDI in clinical trials and how the majority of trials only consider participant recruitment. Further elaboration on why it is important to also consider EDI in the recruitment site specifically would be valuable here. 

 With regard to the data used, it would be useful to provide rationale as to why only "non-white" ethnic groups were included. "Non-white" individuals are not an homogenous group so it might be useful to also consider the use of additional ethnic groups (e.g., the broad census groups) to help determine how to be more inclusive overall. 

 To allow replicability and for interested readers, it would also be useful to have an explicit list or table of the specific variables that were pulled from the datasets (e.g., the proxies or surrogate markers used for the TIDE trial). 

 Lastly, the study provides three great examples on how we can use publicly available datasets to select a diverse recruitment site. Within the limitations/discussion section, it might be useful to discuss the advantages/limitations of using publicly-available datasets compared to large population-level linked datasets which provide data at a person-level, rather than aggregated. 

 Overall, a very thought-provoking article that provides strong justification for using data to improve inclusivity in clinical trials going forward.

Is the rationale for developing the new method (or application) clearly explained?

Partly

Is the description of the method technically sound?

Yes

Are the conclusions about the method and its performance adequately supported by the findings presented in the article?

Yes

If any results are presented, are all the source data underlying the results available to ensure full reproducibility?

Yes

Are sufficient details provided to allow replication of the method development and its use by others?

Partly

Reviewer Expertise:

Child development; cohort studies; linked data

I confirm that I have read this submission and believe that I have an appropriate level of expertise to confirm that it is of an acceptable scientific standard.

# Response

**Published:** 2024-09-07

**Authors:** Alison Booth

**Affiliations:** Health Sciences, University of York, York, England, UK

Thank you for your supportive review and helpful suggestions, all of which we have addressed in Version 2.
